# Sex Differences in Autism Spectrum Disorder: Focus on High Functioning Children and Adolescents

**DOI:** 10.3389/fpsyt.2021.539835

**Published:** 2021-07-09

**Authors:** Concetta de Giambattista, Patrizia Ventura, Paolo Trerotoli, Francesco Margari, Lucia Margari

**Affiliations:** ^1^Child Neuropsychiatric Unit, University of Bari Aldo Moro, Bari, Italy; ^2^Department of Biomedical Sciences and Human Oncology, University of Bari Aldo Moro, Bari, Italy; ^3^Psychiatric Emergencies in Adolescence Unit, University of Bari Aldo Moro, Bari, Italy

**Keywords:** autism spectrum disorder, high functioning, female, gender, sex

## Abstract

Autism Spectrum Disorder (ASD) has historically been studied, known, and diagnosed in males. Females tend to remain unidentified, especially those with average intelligence abilities. This sex/gender difference might be partially explained by biological risk factors, but it is probably also bound to methodological issues. The present study aims to examine phenotypic characteristics (cognitive, emotive, socio-communicative, and academic) of a group of 54 females with ASD matched to a group of 55 males with ASD (3–18 years), all without cognitive impairment. Results suggest that there are subtle, yet potentially meaningful, quantitative, and qualitative phenotypic differences between females and males that common screening tests are not always sensitive enough to recognize. Further studies to improve practice and course for the assessment of females, reducing sex/gender-based inequities in ASD care, are required.

## Introduction

Autism spectrum disorder (ASD) is a complex neurodevelopmental and lifelong disorder. According to the 5th edition of the Diagnostic Statistical Manual of Mental Disorders [DSM-5; (1)], specific diagnostic criteria include impairment in social skills and communication together with restrictive and repetitive behaviors, interests, or activities, as well as sensory abnormalities. In order for a diagnosis to be made, these deficits must be severe enough to significantly impair patients functioning. Although symptoms may be noticed and diagnosed in early childhood, they could also manifest later in life, when social demands and requirements exceed the limited range of functioning of an ASD individual.

In recording procedures of DSM-5, severity should be recorded in terms of the level of support needed, adding the specification of with/without accompanying intellectual and/or language impairment, which should be recorded thereafter. “High” and “low” Functioning Autism are terms, even if not used to identify nosographic categories, commonly used in scientific literature to identify patients diagnosed with ASD according to intellectual abilities (Intelligence Quotient, IQ, respectively higher or lower than 70).

The prevalence of ASD has significantly increased over the past few decades, nowadays affecting 1 in 59 children in the United States, as reported by recent findings from Centers for Disease Control data (2). ASD disproportionately affects males; the over-representation of boys with respect to girls is one of the most replicated findings in the field of ASD. The gender ratio for the spectrum. as a whole is consistently reported at around 4:1 M:F (3–6), ranging according to intelligence quotient (approximately 2:1 in ASD with intellectual impairment; up to 11:1 ASD without intellectual impairment) and age (nearly 5:1 in child and adolescents; around 2:1 in adults) (7–9). Actually, amongst epidemiological surveys screening the general population (non-referred samples), those bearing meaningful outcomes have reported a lower male to female ratio, closer to 2–3:1 (10–12). This evidence suggests that, in addition to biological factors, there could be methodological factors influencing recognition in females. Since the beginning, scientific literature has been male-based: Kanner and Asperger's samples were, respectively, prevalently (8 males in 11 children) or exclusively (4 males) composed by male subjects (13, 14); the vast majority of subsequent research was carried out on male samples; in fact, there were few detailed female-oriented studies. Additionally, they took into account only moderate sample sizes and focused especially on adults with a comorbid intellectual disability or on a limited number of domains (15–17).

Among biological factors, recent data support the existence of sex-linked genetic (e.g., X chromosome gene protective effects) and hormonal factors (e.g., prenatal hormones), which would attenuate risk in females and enhance it in males (4, 9, 18–24). Lai (21, 25) hypothesized that females have a higher genetic vulnerability threshold (higher mutational burden) than males but also that environmental and epigenetic factors can modify the vulnerability in both sexes. According to Lai's theory of higher mutational burden, Bourgeron (26) demonstrated a higher amount of copy number variation and single nucleotide polymorphism in female ASD. Concerning epigenetic factors, different studies supported the hypothesis that there may be a link between ASD or ASD traits and steroid hormones abnormalities, including elevated fetal sex steroids, pubertal hormones' imbalance, and other steroid hormones abnormalities (18, 23, 25, 27, 28). In particular, prenatal testosterone and the whole steroidogenic activity predicts, in both sexes, autistic cognitive-behavioral characteristics (29). The prenatal hormonal environment may sex-specifically cause macroglial activation in the developing brain, potentially following maternal immune activation (25, 30, 31). With regard to environmental factors, it is important to highlight how sociocultural factors have an effect on sex/gender brain differences and similarities: neuroplasticity could explain the way the experience drives and shapes brain development during childhood and adolescence. Conversely, gender may influence how individuals express their autism-related characteristics through intrapersonal processes, family, and social interactions.

Neuroimaging studies show that females and males greatly differ in some aspects of brain development, while in other ones they may be quite similar both for TD and ASD subjects. Normative sex differences have been detected both by neuroimaging studies regarding brain size, volume of defined areas (32), corpus callosum size (33), gray and white matter distribution (34, 35), both by functional imaging research regarding brain intrinsic functional organization (36), connectivity (37–39), and tasks involving empathy, emotions, language, and visuo-spatial processing (40, 41). In a paper analyzing brain volumetry, the temporal lobe was found to be larger in ASD than in TD subjects and in male than in female ASD subjects (32). Sex differences were found between ASD and TD subjects and between male and female ASD subjects also in terms of volumetric characteristics of the corpus callosum (33). Findings from a voxel-based morphometry study (35) revealed that gray and white brain patterns in females with ASD significantly resemble neural masculinization; conversely, in males with ASD, they resemble neural feminization. Consistently, connectivity studies show a pattern of hyper intra-hemispheric connectivity in female ASD subjects (more similar to male TD subjects) as well as a pattern of hypo connectivity in male ASD (more similar to female TD subjects) (42). Nevertheless, results in this field are inconclusive and heterogeneous, also considering variability over time and by age cohorts (43, 44), and the research about differences as well as similarities between sexes keeps being a topic of great scientific interest.

Among methodological factors, diagnostic criteria, tests, and professional's expertise could have a role in females' under-diagnosis, since diagnostic and screening tests of ASD are developed on the basis of the male-typical phenotype, and assessment of females is restricted to areas where they are most similar to males. Therefore, those who do not meet the male-typical behavioral descriptions are likely to be missed (9, 17, 20, 25, 45–52).

In this unclear perspective, the role of sex and gender on the phenotypic expression of ASD should be a focus of research, where the term “sex” refers to “a person's biological status,” while the term “gender” refers to “the attitudes, feelings, and behaviors that a given culture associates with a person's biological sex” (53).

This study aimed to investigate the characteristics of the female ASD phenotype in pediatric and adolescent age, recognizing ASD differences and similarities between females and males, as well as clarifying if a male bias in ASD actually exists in available screening and diagnostic tools and/or in DSM-5 diagnostic criteria.

## Methods

### Participants

Children and adolescents with a diagnosis of ASD were recruited between November 2017 and May 2019 at the Child Neuropsychiatric Unit and at the Adolescence Psychiatric Emergencies Unit of the University of Bari, and they were divided into two groups according to biological sex. In an attempt to create homogeneous groups, we selected the first 100 consecutive patients best matched in age. The final participants included in our analysis were 109, divided into two groups: a group of females (*n* = 54; age range 3–18) and a group of males (*n* = 55; age range 3–18). The inclusion criteria were age below 18 years, being diagnosed with level 1 and 2 ASD according to DSM-5 diagnostic criteria, and an intellectual quotient (IQ) higher than 70. All the subjects were diagnosed according to clinical judgement from the expert team and supported by standardized diagnostic tools specific for ASD such as the Autism Diagnostic Observational Schedule, Second Edition (ADOS-2) (54, 55), Autism Diagnostic Interview-Revised (ADI-R) (56), Childhood Autism Rating Scale (CARS) (57), and Michigan Autism Spectrum Questionnaire (MASQ) (58). In order to screen for other psychiatric disorders, we used medical history, clinical observation, and the children behavioral checklist (CBCL) (59).

All procedures performed were in accordance with ethical standards of the local Ethics Committee of the Azienda Ospedaliero-Universitaria Consorziale Policlinico di Bari and with the 1964 Helsinki declaration and its later amendments. Informed consent from parents and assent from children and adolescents were obtained prior to enrollment.

### Procedure and Materials

The research team, composed by child and adolescent neuropsychiatrists with expertise in the autism field, collected data from anamnestic interview, clinical, and neuropsychological evaluations.

The profile of ASD-specific features was assessed with a clinical evaluation, including behavioral observation and psychiatric interviews, according to DSM-5 ASD diagnostic criteria, and with the administration of screening questionnaires and diagnostic interviews, depending on the age, to caregivers and patients.

*The Autistic Spectrum Quotient Questionnaire* [AQ; (60)] measures the level of autistic traits that an individual may possess. This questionnaire has three versions depending on the age of respondents: aged 4 to 11 (Child version), 12 to 15 (Adolescent version); and 16 and older (Adult version). The Adult version is a self-report questionnaire.

*The Empathy Quotient Questionnaire* [EQ; (61)] measures the cognitive, affective, and behavioral aspects of empathy. This questionnaire has three versions depending on the age of the respondent: aged 4 to 11 (Child version), 12 to 15 (Adolescent version); and 16 and older (Adult version). The Adult version is a self-report questionnaire.

*The Systemizing Quotient Questionnaire* [SQ; (61)] assesses the interest in systems across a range of different classes of systems. The questionnaire has three versions depending on the age of the respondent: aged 4 to 11 (Child version), 12 to 15 (Adolescent version); and 16 and older (Adult version). The Adult version is a self-report questionnaire.

*The Australian Scale for Asperger's Syndrome* [ASAS; (62)] is a questionnaire designed to identify behaviors and abilities indicative of Asperger Syndrome (AS) in children and is divided into five domains (social emotional abilities, communication skills, cognitive skills, specific interests, and movement skills). Each domain consists of different questions, having scores ranging from one (if the indicative behavior is rarely present) to six (if the indicative behavior is frequently present). Additionally, ASAS includes a section called “other characteristics” that investigates the presence/absence of atypical sensitivity, stereotypical movements, and language delay. ASAS results do not yield cut-off scores but give important information for clinical assessment. Responses to each question are in the form of “yes” (positive score) if the behavior is present (ASAS's score 4–6) or “not” (not scored) if it is not (ASAS's score 0–3).

*The Autism Spectrum Diagnostic Interview* [ASDI; (56)] is an investigator-based interview, built around the Gillberg and Gillberg (63) criteria for Asperger syndrome, designed for use by clinicians who want to rapidly determine whether or not an individual patient is likely to meet criteria for a diagnosis of AS or Autism. It comprises six sections: social interactions, narrow interests, routines, language, non-verbal communication, and clumsiness. Each section consists of different questions, whose scores range from one (if the indicative behavior “does not apply”) to three (if the indicative behavior “definitely applies”). The criterion is met when the score indicated for each section is obtained.

The cognitive level was assessed with the Italian version of Wechsler Intelligence Scale for Children, Fourth Edition (WISC-IV, in 59 patients) and Preschool and Primary Scale of Intelligence, Third Edition (WPPSI-III, in 29 patients); Leiter International Performance Scale Revised-Visualization and Reasoning battery (Leiter-R, in 21 patients) was administered, as an alternative to WISC-IV and WPPSI-III, to subjects with verbal disorders; academic achievement was assessed with the following batteries of tests, validated for the Italian language: MT Group Reading Tests for Primary School; MT Group Reading Tests for Middle School; MT Group Advanced Reading and Mathematics Tests for the first biennium of Secondary School; Battery for the Evaluation of Developmental Dyslexia and Dysorthography for Primary and Middle school; and Evaluation Tests of Calculation Ability for Primary School and Evaluation Tests of Calculation Ability and Problem Solving for Middle School.

### Statistical Analysis

Quantitative data were summarized as mean and 95% confidence interval of the mean. To evaluate the difference by sex and level, given the adequate approach to normal distribution, the ANOVA model was applied separately for each quantitative variable (age, intelligence quotient, and AQ, EQ, and SQ scales) as dependent, sex, ASD level, and interaction as effects. *Post-hoc* comparisons were performed by Tukey test. Results were shown as means estimate by the linear model and its 95% confidence interval.

Qualitative variables were summarized as count and percentage. Answers to ASAS, classified in two categories, were analyzed accounting for multiplicity, therefore comparisons of percentage between the independent male and female groups, stratified by level of ASD, were all performed by Fisher exact test, and *p*-values were adjusted with a permutational method. The same procedure was used to analyze results and correct *p*-values for signs and symptoms, school abilities, and comorbidities.

Only adjusted *p* < 0.05 were considered for statistical significance. Analysis was performed with SAS 9.4. The PROC MULTTEST was applied to account for multiple testing.

## Results

### Sample Characteristics

Demographic characteristics of the sample were illustrated, separately for males and females with ASD and for levels 1 and 2 of severity (Table 1).

**Table 1:** Demographic and clinical characteristics of the sample.

	**Total ASD**			**Level 1 ASD**			**Level 2 ASD**		
	**Females**	**Males**	***p*-value**	**Females**	**Males**	***p*-value**	**Females**	**Males**	***p*-value**
***N***	54	55		33	32		21	23	
**Age mean (95% CI)**	10.14 (10.66–12.69)	9.86 (7.08–9.57)	0.7315	12.27 (10.84–13.71)	11.06 (9.61–12.52)	0.6439	8 (6.2–9.79)	8.65 (6.94–10.37)	0.954

*ASD, autism spectrum disorder; CI, confidence interval*.

### First Medical Consultation

Age and reason for first medical consultation were collected and analyzed in relation to sex and level of severity (Table 2).

**Table 2:** Age and Reason for first medical consultation.

	**Total ASD**					**Level 1 ASD**					**Level 2 ASD**				
	**Females**		**Males**		***p*-value**	**Females**		**Males**		***p*-value**	**Females**		**Males**		***p*-value**
**Age of first consultation**	**Mean**	**(95% CI)**	**Mean**	**(95% CI)**		**Mean**	**(95% CI)**	**Mean**	**(95% CI)**		**Mean**	**(95% CI)**	**Mean**	**(95% CI)**	
	6.11	(5.42–6.96)	4.95	(4.39–5.58)	0.0178	9.29	(7.96–10.85)	6.32	(5.39–7.39)	0.0042	4.04	(3.33–4.9)	3.89	(3.23–4.69)	0.9932
**Reasons of first consultation**	***N***	**%**	***N***	**%**		***N***	**%**	***N***	**%**		***N***	**%**	***N***	**%**	
Neurodevelopmental delay	16	29.62	23	41.81	0.2316	2	6.06	6	18.75	0.1487	14	66.67	17	73.91	0.7438
Attention and learning problems	15	27.77	19	34.54	0.5363	15	45.45	17	53.13	0.6228	0	0.00	2	8.70	0.4894
Emotional problems	12	22.22	5	9.09	0.0691	9	27.27	3	9.38	0.1081	3	14.29	2	8.70	0.6575
Behavioral problems	9	16.66	8	14.54	0.7973	5	15.15	6	18.75	0.7508	4	19.05	2	8.7	0.4029
Feeding problems	2	3.7	0	0	0.2431	2	6.06	0	0.00	0.4923	0	0.00	0	0.00	-

*ASD, autism spectrum disorder; CI, confidence interval*.

The mean (95%CI) age of the first medical consultation was statistically significantly higher in the female group than in the male group (*p* = 0.0178), especially in Level 1 (*p* = 0.0042). Females underwent their first medical consultation about 2 years later in the total sample and 3 years later than males in Level 1.

No significant differences were found in reasons for first medical consultation, even if emotional and feeding problems were higher in the female group (respectively, 22.2 and 3.7%), while neurodevelopmental and attention/learning ones were higher in the male group (respectively, 41.81 and 34.54%).

### DSM-5 ASD Diagnostic Criteria

The presence or absence of persistent deficits in social communication and social interaction, as well as restricted, repetitive patterns of behavior, interests, or activities, across multiple contexts, currently or by history, listed as illustrative examples of DSM-5 ASD diagnostic criteria, was collected and analyzed in relation to sex (Table 3).

**Table 3:** Symptoms and signs from DSM-5 diagnostic criteria.

	**Female ASD**		**Male ASD**		***p*-value**
	***N***	**%**	***N***	**%**	
**(A1) Deficit in social-emotional reciprocity**					
Abnormal social approach	34	62.96	39	70.9	0.34363
Failure of normal back-and-forth conversation	44	81.4	48	87.2	0.2999
Reduced sharing of interests, emotions, or affect	41	75.9	41	74.5	0.7999
Failure to initiate or respond to social interaction	36	66.6	36	65.4	0.7372
**(A2) Deficit in non-verbal communicative behaviors used for social interaction**					
Poorly integrated verbal and non-verbal communication	39	72.2	30	54.5	0.9765
Abnormalities in eye contact and body language	42	77.77	45	81.8	0.2923
Deficits in understanding and use of gestures	28	51.8	34	61.8	0.2409
A total lack of facial expressions and nonverbal communication	7	12.96	14	25.45	0.1203
**(A3) Deficits in developing, maintaining, and understanding relationships**					
Difficulties adjusting behavior to suit various social contexts	33	61.1	47	85.4	0.0084
Difficulties in sharing imaginative play	29	53.7	42	76.3	0.0476
Difficulties in making friends	52	96.29	53	96.36	0.6463
Absence of interest in peers	6	11.11	7	12.7	0.5893
**(B1) Stereotyped or repetitive motor movements, use of objects, or speech**					
Simple motor stereotypies, lining up toys, or flipping objects	33	61.1	33	60	0.5456
Echolalia, idiosyncratic phrases	18	33.33	16	29.09	0.8505
**(B2) Insistence on sameness, inflexible adherence to routines, or ritualized patterns or verbal non-verbal behavior**					
Extreme distress at small changes and/or difficulties with transitions	36	66.66	44	80	0.1065
Rigid thinking patterns. greeting rituals	30	55.55	41	74.54	0.0303
Need to take the same route or eat food every day	29	53.7	25	45.45	0.8808
**(B3) Highly restricted. fixated interests that are abnormal in intensity or focus**					
Strong attachment to or preoccupation with unusual objects	15	27.77	16	29.09	0.5899
Excessively circumscribed or perseverative interest	48	88.8	52	94.54	0.1897
**(B4) Hyper- or hyporeactivity to sensory input or unusual interests in sensory aspects of the environment**					
Apparent indifference to pain/temperature	2	3.7	1	1.81	0.8988
Adverse response to specific sounds or textures	36	66.66	41	74.54	0.4451
Excessive smelling or touching of objects	23	42.59	11	20	0.9945
Visual fascination with lights or movement	3	5.55	12	21.81	0.0209

*ASD, autism spectrum disorder*.

Concerning social communication and social interaction among female and male ASD, there were no statistically significant differences in social-emotional reciprocity; instead, there were some statistically significant differences in developing, maintaining, and understanding relationships. In particular, the female group was significantly less compromised than the male group in adjusting behavior to suit various social contexts (*p* = 0.0084) and in sharing imaginative play (*p* = 0.0476).

Concerning restricted, repetitive patterns of behavior, interests, or activities, the female group proved to be statistically less compromised than the male group in insistences of sameness, rigid thinking patterns, and greeting rituals (*p* = 0.0303). No statistically significant differences were found in stereotyped or repetitive motor movements, use of objects, speech, and highly restricted and fixated interests. The female group appeared statistically less compromised in visual fascination with light or movement (*p* = 0.0209).

### AQ, EQ, and SQ Scores

The mean (95%CI) score of AQ-child (*p* = 0.0006) and SQ-child (*p* = 0.0458) were statistically significantly higher in the male group than in the female group; on the contrary, the mean (95%CI) score of EQ-child (*p* = 0.0282) was significantly higher in the female group than in the male group (Table 4). Lateness in acquiring speech is 18.1% in females with ASD and 46% in males with ASD. No statistically significant differences were found in AQ, EQ, and SQ-adolescents. Females did not exceed the cut-off for either AQ-child or AQ-adolescent. The only difference, found in sub-group of the level of severity, was AQ-child in level 2, significantly lower (*p* = 0.0275) in females (56.6; 95% CI 44.04–69.16) than in males (81.41; 95% CI 69.61–93.21).

**Table 4:** Autism quotient, empathy quotient, systematizing quotient.

	**Female ASD**	**Male ASD**	**p value**
AQ 4–11	57.16 (48.12–66.19)	79.37 (71.14–87.60)	0.0006
AQ ≥ 12	26.03 (20.47–31.59)	29.67 (23.87–35.46)	0.3658
EQ 4–11	28.73 (25.20–32.26)	23.32 (30.05–26.59)	0.0282
EQ > 12	24.61 (19.92–29.30)	26.28 (21.43–31.14)	0.6188
SQ 4–11	18.96 (16.26–21.67)	22.7 (20.22–25.23)	0.0458
SQ > 12	46.08 (37.91–54.26)	36.70 (28.24–45.17)	0.1151

*ASD, autism spectrum disorder; AQ, autism quotient; EQ, empathy quotient; SQ, systematizing quotient*.

### ASAS Score

Among social emotional abilities, unawareness of social conventions or codes of conduct (*p* = 0.0032) and indifference to peer pressure (*p* = 0.001) were significantly less compromised in the female group than in the male group (Table 5).

**Table 5:** Core clinical features obtained from ASAS.

	**Female ASD**		**Male ASD**		***p*-value**
	***N***	**%**	***N***	**%**	
**Social emotional abilities**					
Unawareness of the unwritten rules of social play	9	20.45	24	53.3	0.0032
Avoid social contact	9	20.45	14	31.11	0.5043
Unawareness of social conventions or codes of conduct	14	31.81	24	53.3	0.0847
Lack of empathy	11	25	20	40.44	0.1729
Expecting other people to know own thoughts	12	27.27	21	45.66	0.686
Excessive amount of reassurance	22	50	29	64.44	0.5627
Lack of subtlety in expression of emotion	17	38.6	23	51.11	0.5843
Lack precision in expression of emotion	14	31.8	20	44.44	0.5022
Not interested in competitive sports and activities	18	40.9	18	40	0.7163
Indifferent to peer pressure	10	22.72	28	62.22	0.001
**Communication skills**					
Literal interpretation of comments	18	40.9	29	64.44	0.0631
Unusual tone of voice	11	25	23	51.11	0.0436
Less interested in the other part of conversation	11	25	27	60	0.0016
Less eye contact when in a conversation	11	25	25	55.55	0.0081
Over-precise or pedantic speech	10	22.72	22	48.88	0.0701
Problems repairing a conversation	13	29.54	28	62.22	0.0075
**Cognitive skills**					
Reading books primarily for information	7	15.9	17	37.77	0.1189
Exceptional long-term memory for events/facts	23	52.27	34	75.55	0.551
Lack of social imaginative play	10	22.72	16	35.55	0.708
**Specific interests**					
Fascinated by a particular topic	16	36.36	27	60	0.1612
To be unduly upset by changes in routine	17	38.63	26	57.77	0.2325
To develop routines/rituals	12	27.27	24	53.33	0.0098
**Movement skills**					
Poor motor coordination	10	22.72	16	35.55	0.3685
Odd gait when running	7	15.9	17	37.77	0.1189
**Other characteristics**					
Unusual fear/distress due to ordinary sound	12	27.27	13	37	0.7046
Unusual fear/distress due to light touch on skin or scalp	6	13.63	8	17	0.1469
Unusual fear/distress due to wear particular items of clothing	6	13.63	16	35	0.0099
Unusual fear/distress due to unexpected noises	23	52.27	18	40	0.419
Unusual fear/distress due to see certain objects	3	6.8	9	20	0.3395
Unusual fear/distress due to noisy, crowded places	11	25	22	48	0.0091
A tendency to flap or rock when excited/distressed	14	31.8	19	42	0.7343
Lack of sensitivity to low level of pain	9	20.4	11	24	0.9637
Late in acquiring speech	8	18.1	21	46	0.3333
Unusual facial grimaces or tics	14	31.8	20	44	0.1763

*ASAS, Australian scale for asperger's syndrome; ASD, Autism spectrum disorder*.

Among communication skills, unusual tone of voice (*p* = 0.0436), interest in another part of the conversation (*p* = 0.0016), less eye contact (*p* = 0.0081), and problems reestablishing a conversation (*p* = 0.0075) were significantly less common in the female group than in the male group.

Among specific interests, developing routines/rituals was significantly lower in the female group than in the male group (*p* = 0.0098).

With regard to atypical sensitivity, unusual distress for wearing particular items of clothing (*p* = 0.0099) and too noisy and crowded places (*p* = 0.0091) were significantly lower in the female group than in the male group.

Considering the differences between females and males of the same level of severity, the significance was prevalently confirmed in level 1, except for rituals and distress in wearing particular clothing. Female ASD appear significantly less compromised in expecting other people to know their own thoughts (F 22.58 vs. M 55.17%, *p* = 0.0161) and in lack of social imaginative play (F 16.67 vs. M 44.83%, *p* = 0.0251) exclusively in level 1, but not in the total sample.

### ASDI Scores

The percentage of females with ASD with “speech and language problems” is 75, whiles is 82.22 for males with ASD (Table 6). Non-verbal communication problems were statistically lower in the female group compared to the male group (*p* = 0.0227). Considering differences between females and males of the same level of severity, the difference in non-verbal communication was exclusively confirmed in level 1 (F 61.29 vs. M 93.10%, *p* = 0.023).

**Table 6:** Core clinical features obtained from ASDI scores.

	**Female ASD**		**Male ASD**		***p*-value**
	***N***	**%**	***N***	**%**	
Severe impairment in reciprocal social interaction	35	79.54	38	84.44	0.3725
All absorbing narrow interest	35	79.54	38	84.44	0.3725
Imposition of routines and interests	31	70.45	38	84.44	0.0919
Speech and language problems	33	75	37	82.22	0.2837
Non-verbal communication problems	32	72.72	41	91.11	0.0227
Motor clumsiness	28	63.63	31	68.88	0.3822

*ASD, autism spectrum disorder*.

### Intelligence Quotient

The mean IQ in females with ASD is 108.14, whiles the mean IQ in males with ASD is 104.14, and there were no statistically significant differences (Table 7). There was no significant difference between the two groups in FSIQ and in all the IQ sub-values, except to VIQ of WPPSI-III, which resulted statistically significantly higher in the female group than in the male group (*p* = 0.0202). Considering differences between females and males with the same level of severity, VIQ of WPPSI-III resulted statistically higher in females than males exclusively in level 2 (F 115.33 v. M 87.00; *p* = 0.0108).

**Table 7:** Intelligence quotient.

	**Female ASD**	**Male ASD**	***p*-value**
**Leiter-R (*N* = 21), WPPSI-III (*N* = 29), WISC-IV (*N* = 59)**	**Mean (95% CI)**	**Mean (95% CI)**	
Full scale intelligent quotient	108.14 (1,003.39–112.88)	104.14 (99.29–108.98)	0.2397
**WPPSI-III (*****N*** **=** **29)**			
Verbal intelligent quotient	114.86 (105.31–124.42)	101.45 (95.73–107.17)	0.0202
Performance intelligent quotient	111.70 (102.18–121.22)	107.95 (102.26–113.65)	0.4933
Processing speed quotient	109.25 (92.69–125.81)	98.96 (90.92–107.01)	0.2547
**WISC-IV (*****N*** **=** **59)**			
Verbal comprehension index	112.69 (103.81–121.58)	112.10 (103.89–120.31)	0.9214
Perceptual reasoning index	108.97 (101.80–116.14)	111.97 (105.35–118.59)	0.5405
Working memory index	95.92 (88.68–103.16)	87.16 (80.47–93.85)	0.0805
Processing speed index	83.08 (77.09–89.07)	87.11 (81.58–92.64)	0.3264

*ASD, autism spectrum disorder; Leiter-R, leiter international performance scale-revised; WPPSI III, Wechsler pre-school and primary scale of intelligence-III; WISC-IV, Wechsler intelligence scale for children-IV; CI, confidence Interval*.

### School Learning Abilities

School learning abilities were compared and analyzed in relation to sex and level of severity (Table 8).

**Table 8:** School learning abilities.

	**Female ASD**		**Male ASD**		***p*-value**
	***N***	**%**	***N***	**%**	
Specific learning disabilities	19	52.78	16	47.06	0.7633
Writing	11	30.56	14	41.18	0.2492
Reading	9	25.00	6	17.65	0.8509
Mathematics	16	44.44	12	35.29	0.8473

*ASD, autism spectrum disorder*.

By analyzing the subsample of 70 school-aged subjects, no statistically significant differences were found in learning difficulties between females and males.

### Comorbidities

There were no significant differences in comorbidities between the female and male groups, although a non-significant trend was noticed for anxiety, depression, and eating disorders, which were higher in the female group (Table 9).

**Table 9:** Comorbidities.

	**Female ASD**		**Male ASD**		***p*-value**
	***N***	**%**	***N***	**%**	
At least one comorbidity	49	90.7	47	85.45	0.9350
Motor disorder	21	38.8	20	36.3	1
Attention deficit hyperactivity disorder	38	70.37	41	74.54	0.999
Psychotic disorder	3	5.55	0	0	1
Bipolar disorder	3	5.55	0	0	1
Depressive disorder	11	20.3	7	12.72	1
Anxiety disorder	20	37.03	10	18.1	1
Obsessive compulsive disorder	2	3.7	1	1.8	1
Eating disorder	7	12.96	2	3.6	1
Gender dysphoria	1	1.85	0	0	1
Oppositional defiant disorder	2	3.7	1	1.8	1
Personality disorder	3	5.95	0	0	1

*ASD, autism spectrum disorder*.

## Discussion

The male bias in ASD conceptualization has led to females with ASD being under-studied.

The poor awareness of female ASD phenotype, especially when at higher functioning levels, is common even amongst professionals, and produces frequent under/misdiagnosis and consequent under-treatment, causing persistent confusion and suffering for female individuals in the autistic spectrum (15, 64–67).

Previous studies about sex/gender differences reported contrasting and not conclusive results, probably due to under-sampling and to a lack of homogeneity for age and IQ in the selected population.

This study attempts to characterize HF female ASD phenotype in pediatric and adolescent age, comparing a group of females with a group of males with a diagnosis of ASD, all without intellectual impairment.

Our aims were, first of all, to evaluate the potential sex/gender differences in clinical phenotype (core autistic domains, cognitive functioning, and co-occurring conditions) and in developmental trajectories between males and females with ASD, and secondly, to examine if a male bias exists in the available tools and in ASD DSM-5 criteria based on matching clinical findings of the expert team and scores obtained from different screening questionnaires and diagnostic interviews.

The presented results have been analyzed based on biological sex. The role of gender identity on clinical expression was not addressed in this study (even more so considering the low average age of the sample). Nevertheless, according to previous research (25), given the complexity in distinguishing the effects of sex and gender on ASD expression, especially during early pediatric age, in this study we used the term “sex/gender” to express the overlap between them.

### Social-Communication Abilities

Differences in social-communication abilities between male and female children and adolescents with ASD have been reported in several previous studies, especially within ASD without intellectual impairment (25, 47, 48, 52, 68); conversely, other studies highlighted sex/gender similarities (69–72).

In this study, males and females with ASD, assessed with psychiatric interview according to DSM-5 criteria and illustrative examples, globally presented similar deficits in social communication and interaction, even if females with ASD showed greater capacity in developing, maintaining, and understanding relationships and, in particular, in adjusting behavior to suit various social contexts. This difference was also obtained from parental reports, assessed by the administration of screening questionnaires to caregivers (ASAS) in which results showed that, statistically, females are significantly better in terms of awareness of social conventions or codes of conduct.

Indeed, since Wing's hypothesis (73), until recent literature studies (15, 25, 62, 66, 68, 74–77), these relative strengths in females with ASD could reflect coping mechanism that allows them to camouflage themselves into society. “Camouflaging” is the tendency to cope (consciously or unconsciously mimicking or learning) through conventional and acceptable behaviors and strategies in social situations. Being often good observers, females with ASD could copy strategies from peers to be what the counterpart expects; nevertheless, given the artificiality of this effort, they sometimes could have difficulties understanding different types and grades of relationships, modulating behaviors, and managing conflicts. This exhausting attempt to adapt or copy other one's behaviors can bring to a sensation of losing or not developing their own identity, thus, could increase the risk of stress and unhealthy relationships, as seen from other studies (64, 78, 79).

All these social variables may be difficult for professionals to recognize (even because shyness is considered a typical pattern in cultural ideas of females), so their uncomfortable feelings are often misconstrued.

According to clinicians' reports, there were no statistical differences in “abnormalities in eye contact” between males and females with ASD. According to parents' reports, males with ASD made significantly “less eye contact when in a conversation.” This seemingly conflicting result could be explained by the difference between the wording of the illustrative example in DSM 5 criteria and the one used in questionnaires for parents (ASAS). As a matter of fact, females, rather than avoiding eye contact, often present an excessive eye-fixation (called hyper-fixation) with an abnormal, not-modulated gaze that might be mistaken to an untrained eye, according to previous literature (80, 81). Our findings converge on the idea, previously argued in the literature, that socio-cultural constructions of gender and understandings of ASD may contribute to under- or misrecognition of ASD in females.

### Sameness and Sensitivity

In the present study, stereotyped or repetitive motor movements and speech, as well as extremely restricted and repetitive interests, were equally present in males and females; instead, as found from clinical findings as well as screening and diagnostic tools, the inflexible adherence to routines or ritualized patterns were statistically less common in females. This result is in accordance with previous literature which found sex/gender differences of high-functioning population prevalently in the so-called “higher-order RRBs” (that is, complex behaviors, insistence on sameness and routine and in-depth circumscribed interests) rather than in “lower-order RRBs” (that is, simple stereotyped motor movements and speech) which were often equivalent in both sexes/genders, in preschoolers and lowest functioning (70, 82, 83). In addition to the “order of RRBs,” the “type of interests and RRBs” is another point of divergence (69, 82, 84). As emerged from our clinical evaluation, even if not captured by measurement tools, the type of interests in females could appear less autistic-like (i.e., trains, numbers, and videogames), and more similar to those of their neurotypical peers (i.e., dolls, animals, horses, celebrities, fashion, make-up, arts, books, and manga).

Atypical responses to noise, texture, smell, visual stimuli were not included in diagnostic criteria from the previous edition of DSM; they appear in the last version of the Manual, within criterion B, with probable positive implications on females with ASD diagnosis. Previous research reported more sensory symptoms in females with ASD than in males with ASD (52). In the present study, females proved to be less sensitive to lights and, from parents' questionnaires, to noises and particular clothing.

Since the vast majority of available screening and diagnostic scales, including those selected in this study, do not comprise either a focus on interests and stereotypes or a focus on sensitivities (or, in any case, it does not include all the sensitivities), and considering the impact that these aspects could have in ordinary life, clinicians must carefully analyze this aspect throughout oriented questions with the aim to avoid underestimation of these features.

### Cognitive Functioning

As for the domain of cognitive functioning, previous investigations have found both similar (85, 86) and divergent (87, 88) cognitive abilities between males and females with ASD. In this study, FSIQ and subitems were not statistically different, except for verbal and language abilities in preschoolers (VIQ from WPPSI-III), resulting in better scores in females. The delay in acquisition of language is more common in males, as seen in clinical experience as well as in this study; this could explain the better verbal competence of preschool females. Moreover, this finding possibly reflects the sex difference also observed in language for neurotypically developing males and females (16).

### Comorbidities and Developmental Trajectories

According to recent literature (89–91), in the present study, both males and females presented a high percentage of co-occurring conditions (between 85.45 and 90.7 %). The most frequent comorbidity in both groups was ADHD, co-occurring in about 70% of cases, without significant differences between males and females. This evidence differs from previous literature in which ADHD comorbidity appeared more common in males (92, 93). In accordance with other authors, we hypothesized that the diagnosis of ADHD was underrated in females since females with ADHD have different, more hidden, symptomatology, showing inattentive rather than hyperactive manifestations (16, 94). Concerning internalizing disorders, our findings were different from previous literature reporting a statistically significant prevalence of internalizing disorders in females, in that we rather found a non-significant trend for anxiety, depressive, and eating disorders in the female group (95–100). The mean age of the sample (10 years old) could justify these findings since internalizing and feeding disorders often occur in adolescence or adulthood. In this study we found that caregivers ask for a first specialist medical consultation at different ages and for different reasons: females were referred later to clinical services, particularly for level 1 ASD (on average 3 years later). This finding fits with previous literature (7, 25, 67, 101, 102), also showing how females who received an early diagnosis of ASD often have intellectual impairment.

### Diagnostic Tools and DSM-5 Criteria

Scores from screening and diagnostic tools, selected in this study, reveal some inconsistencies between the results obtained from questionnaires administered to parents (ASAS and Baron Cohen Questionnaires, i.e., the AQ, EQ, and SQ), showing greater differences between males and females with ASD, and interviews carried out by clinicians (according to DSM-5 criteria and ASDI), showing more homogeneous results. These inconsistencies could be explained by the different perceptions of clinicians in respect to parents who may underestimate or not recognize some characteristics as atypical.

In the present study, higher scores from AQ and SQ and lower ones from EQ were found in male children, with a reduction of this gap in adolescence. Population surveys on autistic traits confirmed that males have more autistic traits than females measured by Baron Cohen Questionnaires (AQ, EQ, and SQ), with attenuation in adulthood (103). Even if it is not a focus of this study, the adult attenuation of sex/gender differences could be linked with a greater insight of females growing up, as well as a decreased number of adult males that received the diagnosis. Moreover, finding specific female items or examples could be challenging, even in Baron Cohen Questionnaires.

In this study, applying examples listed in DSM-5 criteria, no sex differences were found in the broad social-communicative and sameness criteria; nonetheless, numerous differences were evident in how boys and girls come to meet each criterion. Specific sex/gender examples could be included in diagnostic criteria to detect these qualitative differences.

## Conclusion

Results suggest that there are subtle, yet potentially meaningful, quantitative and qualitative ASD phenotypic differences between males and females that common screening tests are not always sensitive enough to recognize. Thus, a sex/gender disparity, in terms of identification and support, persists, above all within high functioning ASD. It might result in HFA females not receiving much-needed diagnosis and treatment, with a negative effect on their mental health and well-being. In order to prevent the risk of late- or misdiagnosis, diagnostic criteria, and tools could benefit from the addition of specifiers and female-oriented examples. In this way, all the clinicians, even with different expertise, could benefit from more sensitive measures to detect females' characteristics. This could have a positive impact on the diagnostic process, health services, and research paths.

## Limitation and Future Direction

This study has two levels of limitations: the lack of a female-male neurotypical control group and the wide age range.

The absence of a control group might overrate some sex/gender differences as specific to ASD instead of normative sex differences. One of these could be females' better communicative development with respect to males, which is also common in the neurotypical population.

Having a wide age range (3–18 years), the sample does not allow consideration of sex/gender differences in infancy and adolescence. Previous findings show that sex/gender differences are less evident in toddlerhood and more evident in adolescence, both in core features and in comorbidities (16, 104). In our study, these age-related differences are just mentioned but not examined in depth. For these reasons, drawing sums on a limited sample of patients can be speculative.

Future directions should involve enrolling a larger sample, exploring sex/gender differences matching with TD subjects, and differentiating for age. Moreover, it could be useful to develop and test measures (illustrative examples and tools) that capture female as well as male manifestations of ASD.

## Data Availability Statement

The datasets analyzed in this article are not publicly available. Requests to access the datasets should be directed to Concetta de Giambattista, concettadegiambattista@gmail.com.

## Ethics Statement

All procedures performed were in accordance with ethical standards of and approved by the Ethics Committee of the Azienda Ospedaliero-Universitaria Consorziale Policlinico di Bari and with the 1964 Helsinki declaration and its later amendments. Written informed consent from parents and assent from children and adolescents were obtained prior to enrollment.

## Author Contributions

CdG and PV equally contributed in designing the study. LM coordinated the study. CdG, PV, and FM participated in data collection. PT performed data analysis. All authors participated in data interpretation and helped to draft with a comorbid intellectual the manuscript.

## Conflict of Interest

The authors declare that the research was conducted in the absence of any commercial or financial relationships that could be construed as a potential conflict of interest.
